# Exploring the choices and outcomes of older patients with advanced kidney disease

**DOI:** 10.1371/journal.pone.0234309

**Published:** 2020-06-10

**Authors:** Rhodri Pyart, Justine Aggett, Annwen Goodland, Hayley Jones, Alison Prichard, Julia Pugh, Nerys Thomas, Gareth Roberts

**Affiliations:** Department of Nephrology and Transplantation, University Hospital of Wales, Cardiff, United Kingdom; Kaohsiung Medical University Hospital, TAIWAN

## Abstract

A lack of data on patient choices and outcomes at the time of pre-dialysis planning limits meaningful shared decision making, particularly in older frailer patients. In this large retrospective cohort study of patients aged over 70 seen by the pre-dialysis clinic (2004–2016) of a large single centre in the United Kingdom (1,216 patients), age, sex, comorbidity, poverty and frailty were used to predict choice of renal replacement therapy (RRT) over maximum conservative management (MCM). The impact of patient choice of RRT versus MCM was used to predict survival from the time of choice using multivariable Cox proportional hazards regression. Older age, female sex, greater poverty and greater frailty were associated with choosing MCM, whilst comorbidity had no significant impact on choice. At 5 years of follow up, 49% of all patients had died without receiving RRT. Over 70% of the patients choosing MCM died with better kidney function than the median level at which those starting RRT initiated treatment. Frailty and age were better predictors of survival than comorbidity and in patients with at least moderate frailty, RRT offered no survival benefit over MCM. In conclusion, analysing outcomes from the time of choice may improve shared decision making. Frailty should be routinely assessed and collected and further work may help predict which patients are unlikely to survive or progress to end stage renal disease and may not need to be burdened with making a pre-dialysis choice.

## Introduction

Pre-dialysis education programmes are now in widespread use across the renal community [1, 2]. Informing patients through a shared decision-making process can influence patient treatment decisions and improve outcomes [3, 4]. Shared decision making must be underpinned by robust outcome data presented in a way that is meaningful to patients and carers [5–8].

Currently, most outcome studies and registry data have focussed on what happens to patients from the time of commencing renal replacement therapy (RRT) resulting in a paucity of data on the outcomes of patients who have made a choice but not yet started treatment or chosen maximum conservative care (MCM) [9–11]. Given that many, older and frailer patients who enter pre-dialysis programmes never actually start RRT, it is vital that we have a better understanding of what happens to these individuals from the time of modality choice (as well as from the time of modality start) [12–16].

Here we sought to describe the choices and outcomes of a large cohort of older pre-dialysis patients who underwent pre-dialysis education in our centre.

## Materials and methods

### Patient population

A retrospective analysis was undertaken on 1,216 consecutive patients aged 70 years and older who had been referred for pre-dialysis education over a twelve-year period (2004–2016). In line with national guidelines, all patients with progressive renal failure in our unit are referred for pre-dialysis education when their eGFR is persistently below 20ml/min [1]. The pathway followed by patients is detailed in S1 Fig. The MCM pathway takes a holistic approach involving members of the multi-disciplinary team to help maintain patient quality of life and wellbeing while coordinating their care with community physicians, geriatricians and palliative care colleagues to minimise the burden of ongoing management.

Our centre provides tertiary renal care for a population of 1.4 million people in a densely populated region that includes some of the most socioeconomically impoverished areas in western Europe [17]. The population is ethnically overwhelmingly white European.

### Data collection

The pre-dialysis education programme in our unit consists of a structured medical, functional and psychosocial assessment of the patients’ circumstances, needs and priorities. This is usually undertaken by a specialist nurse in the patients’ own homes with the overall aim of finding a treatment that best meets a patient’s values and preferences. A comprehensive home visit report is produced after the home visit (S2 Fig) and key aspects including modality choice are stored in a bespoke renal database. The patient, family, specialist nurse and clinicians continue to regularly meet in a clinic setting in a shared decision making process. If patients change their mind during this time, both their initial and final choice are recorded in the database.

This database also contains information on patient demographics and comorbidity, laboratory results, treatment modality and date of death. The Welsh Index of Multiple Deprivation was used to map patient post codes to small areas (LSOAs = lower-layer super output areas) of relative poverty (“deprivation” in the UK) [18]. Each LSOA has an average population of 1,600 people and the catchment population is made up of 851 LSOAs in South East Wales. The WIMD ranks the LSOAs from 1 to 851 with 1 being the poorest area.

The Charlson Comorbidity Index (which has been validated in end stage renal disease (ESRD)) was used to calculate a weighted score of comorbidities [19, 20].

In a subset of 486 more recent patients who underwent pre-dialysis education (and who all had comorbidity data), an electronic report was accessible which contained adequate information to be able to retrospectively estimate a Clinical Frailty Scale (CFS) score for each patient [21]. This measure of frailty has been previously shown to be an independent predictor of outcomes in dialysis patients [22, 23]. This was treated as a continuous variable. Frailty was also coded as a categorical variable with a CFS of 6 or higher, defined as patients needing help with all outside activities, equating to at least moderate frailty.

### Outcomes

We aimed to explore how patient characteristics influenced whether patients chose to receive RRT (in-centre haemodialysis (HD), peritoneal dialysis (PD), home haemodialysis (HHD) or pre-emptive transplant) or opted for MCM. The impact of patient characteristics and treatment decisions on survival were compared from the time of final choice.

### Statistical analysis

Data were extracted and anonymised prior to analysis. Statistical analysis was performed using SPSS version 23. Normally distributed data are presented by the mean and standard deviation while for skewed data, the median and inter-quartile range are shown. Categorical data were depicted in percentages. Differences between groups were tested using Students t-test, chi-squared and Mann-Whitney test as appropriate. Kaplan-Meier method was used to derive survival curves and log rank test analyses to compare survival between groups. The impact of individual variables on patient choice and survival were assessed using binomial regression and Cox proportional hazard models respectively. Log minus log survival function plots were used to check the assumption of proportionality.

### Ethics statement

Data was fully anonymised prior to being accessed for use in this service evaluation study. Ethical approval was not required based on the Health Research Authority Research Ethics Committee online assessment tool.

## Results

### Patient demographics

Of the 1,216 patients, the median age at the first pre-dialysis visit was 78 years (IQR 74–83), 61.7% were male, median eGFR 15 ml/min (IQR 13–19) and median CCI comorbidity score was 4 (IQR 3–6) (Table 1).

**Table 1:** Demographics of older patients choosing RRT and MCM.

	Overall cohort, Age ≥ 70, (n = 1 216)	RRT, (n = 841)	MCM, (n = 375)	P values (RRT vs MCM)
**Age at visit (years), median (IQR)**	78 (74–83)	76 (73–80)	83 (79–86)	p < 0.001
**% Male**	61.7%	64.0%	56.4%	p = 0.014
**eGFR at visit (ml/min), median (IQR)**	15 (13–19)	16 (13–19)	15 (13–19)	p = 0.981
**CCI Comorbidity Score, median (IQR)**	4 (3–6)	5 (3–6)	4 (3–5)	p = 0.087
**CFS Score (IQR), (% CFS ≥ 6), n with CFS**	5 (4–6), (29%), n = 486	5 (4–5), (20%), n = 288	5 (4–6), (42%), n = 198	p < 0.001

**Fig 1:**
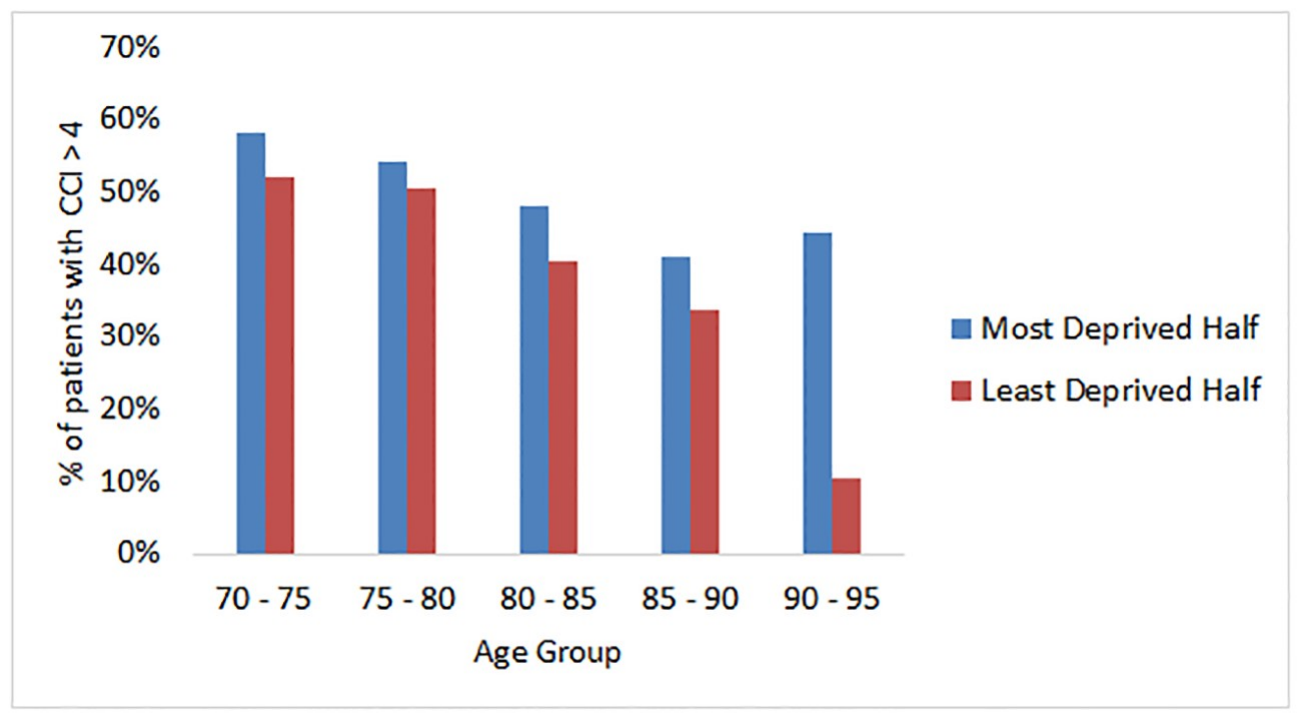
Distribution of patients by age, poverty and higher comorbidity.

Increasing age was weakly correlated with significantly lower levels of poverty (r = -0.078, p = 0.007). Across all age groups, patients from the poorest areas were more likely to have a higher comorbidity score (r = -0.098, p = 0.001) Fig 1. However, comorbidity score reduced with age, likely reflecting a survivor effect (r = -0.150, p<0.001).

### Patient choice

**Fig 2:**
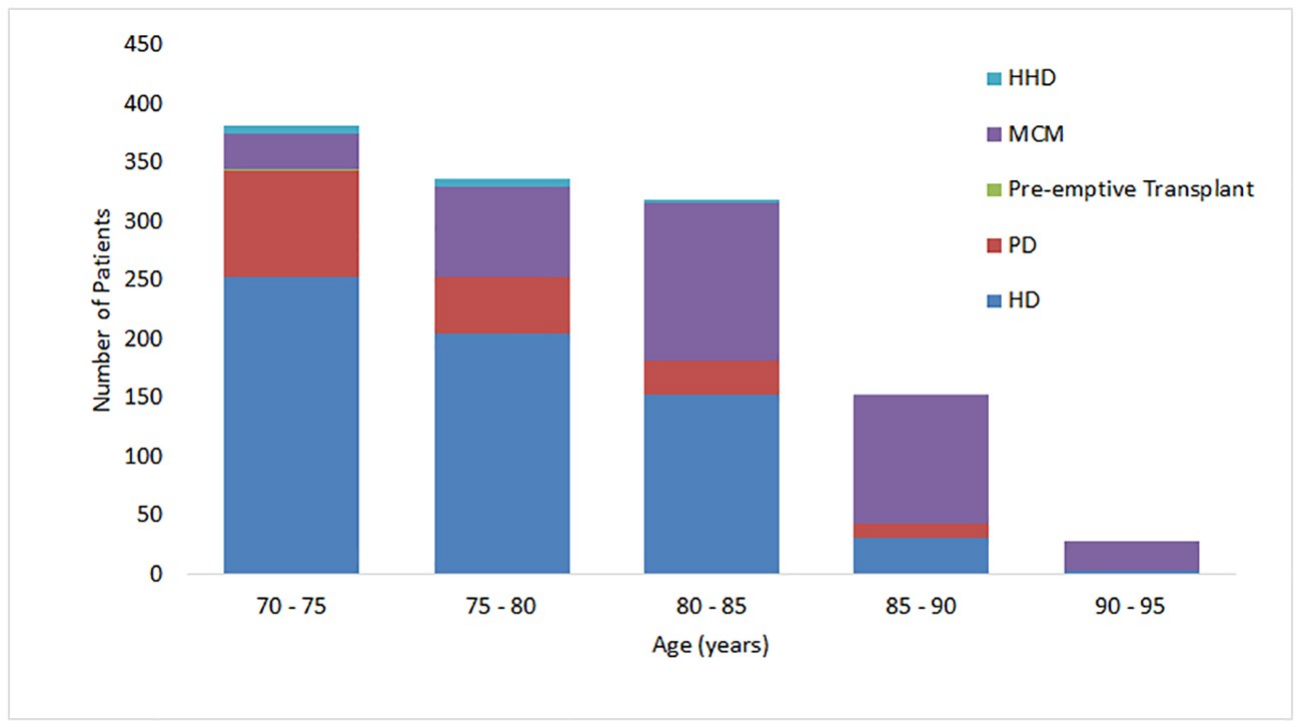
Patient choices after pre-dialysis education.

As shown in Fig 2 the choice of modality varied with age with older patients more likely to choose MCM over RRT (r = 0.481, p<0.001) but less likely to choose PD as their RRT modality (r = 0.180, p<0.001). Overall nearly one third of patients opted for MCM while most of the remainder chose unit HD.

Only 70 (5.8%) of patients changed their mind during the process of pre-dialysis education prior to making a final choice—29 patients switched to MCM from choosing HD/PD and 25 changed from choosing PD to HD. Only 8 (0.7% of the overall total) patients changed from MCM to RRT (all to HD).

The demographics of the patients divided between those choosing RRT or MCM are shown in Table 1. A subgroup of each modality had sufficient information available to estimate a Clinical Frailty Score. Of note, the cohort choosing MCM were older with higher frailty scores. Patients choosing MCM tended to have lower CCI scores, reflecting older patients having less comorbidity, but this difference did not reach statistical significance.

A multivariable logistic regression was performed to ascertain associations between age, comorbidity, sex and poverty and the likelihood of patients choosing RRT over MCM. Older age, being female and being poorer were associated with choosing MCM whilst levels of comorbidity did not impact on choice. In subgroup analysis, CFS was significantly associated with increasing age, however poverty score and sex were no longer significant while CCI remained non-significant (Table 2). Frailty as a categorical variable (CFS <6 vs CFS ≥6 –level chosen as it is a marker of at least moderate frailty and dependency) gave the same result.

**Table 2:** Choice of RRT over MCM.

All ≥ 70; n = 1 216	β	P-value	Odds Ratio	95% CI for Odds Ratio
Sex (Female = 1)	-0.33	0.025	0.72	0.54–0.96
CCI (per unit increase)	-0.07	0.12	0.93	0.86–1.02
Poverty (per unit increase)	0.00	0.004	1.00	1.00–1.00
Age (per year increase)	-0.24	<0.001	0.79	0.77–0.82
≥ 70 with CFS; n = 486				
Sex (Female = 1)	0.10	0.68	1.10	0.69–1.75
CCI (per unit increase)	0.10	0.16	1.10	0.96–1.26
Poverty (per unit increase)	0.00	0.13	1.00	1.00–1.00
Age (per year increase)	-0.23	<0.001	0.79	0.75–0.83
CFS (per unit increase)	-0.48	<0.001	0.62	0.51–0.75

### Transition from choice to event

**Fig 3:**
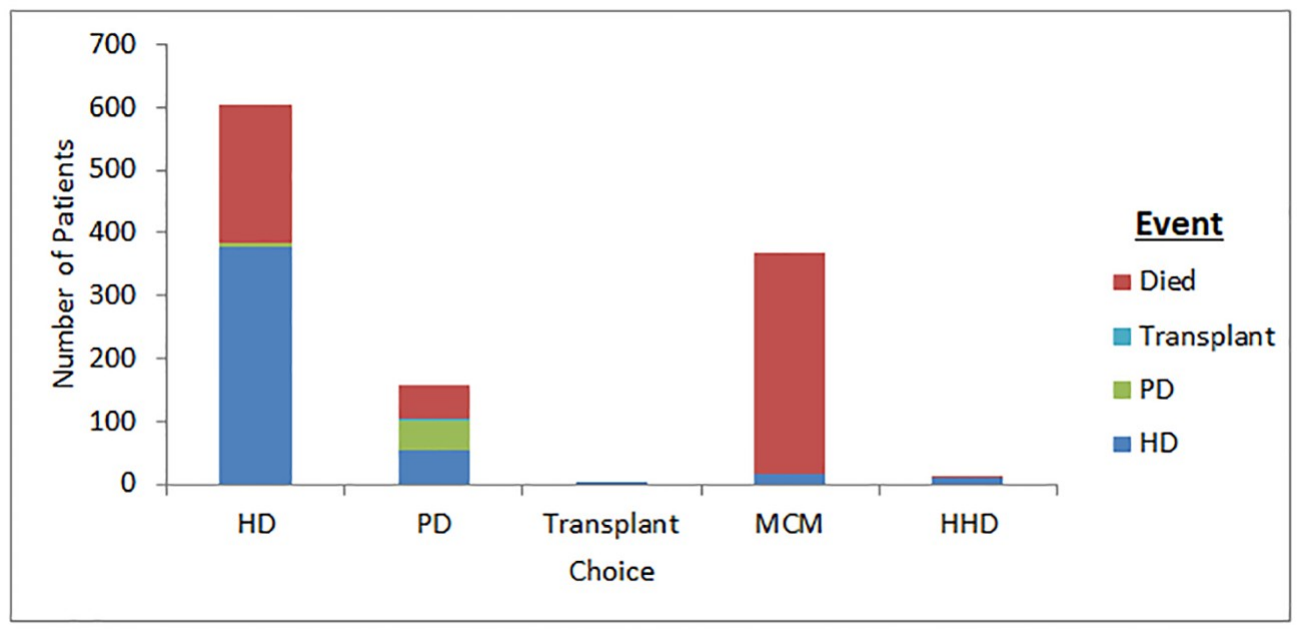
First event where event occurs.

To explore whether patients started the modality of their choice, a different modality or died first, a timeline of a first event of RRT modality or death were recorded for the 86% of patients in whom an event occurred during follow-up (Fig 3).

One third of patients choosing RRT (HD or PD) died before ever starting dialysis (34.8% (HD) vs 32.0% (PD), p = 0.29) Patients who choose PD were less likely to start the modality of their choice compared to those choosing HD (31.3% vs 64.3%, p<0.001). Of the 490 patients who started RRT—only 14 received transplants during follow-up.

### Timelines & all cause mortality

A timeline of 3-year and 5-year survival post choice is shown in Table 3. At 5 years, nearly half of all patients had died without receiving RRT with only 14% of the entire cohort alive on RRT at 5 years. The median unadjusted survival in the MCM and RRT groups was significantly different, at 742 days (95% CI: 637–847) and 1474 days (1,321–1,627) (p < 0.001) respectively.

**Table 3:** Outcomes of older patients at 3 and 5 years from the time of choice.

***n = 1 086***	***Status at 3 Years from Choice***			
**Choice**	**Alive on RRT**	**Alive no RRT**	**Dead Post RRT**	**Dead no RRT**
RRT	31%	28%	19%	21%
MCM	1%	30%	2%	67%
**Total**	22%	29%	14%	36%
***n = 1 005***	***Status at 5 Years from Choice***			
**Choice**	**Alive on RRT**	**Alive no RRT**	**Dead Post RRT**	**Dead no RRT**
RRT	21%	15%	33%	30%
MCM	1%	12%	2%	85%
**Total**	14%	14%	23%	49%

**Fig 4:**
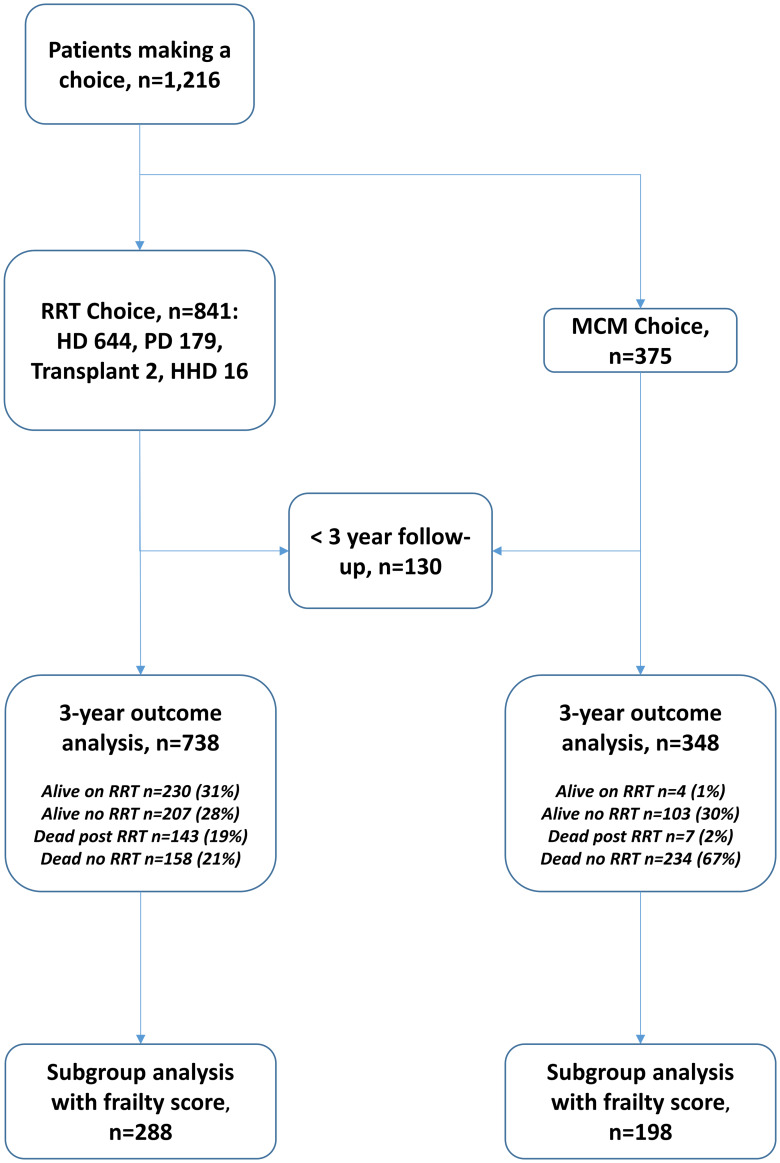
Flowchart of patients and outcomes.

The timeline of events is demonstrated in Fig 4.

In our cohort, the median eGFR at which dialysis was initiated was 9ml/min in keeping with the UK national average mean start eGFR of 8.5ml/min [24]. There was a correlation between higher start eGFR and older age (p = 0.02). There was no survival difference from the time of choice between those who started dialysis with an eGFR above or below 9ml/min. Nearly 70% of patients who chose MCM and died never reached an eGFR of less than 9ml/min suggesting that the majority patients who opt for MCM do not progress to a stage where they would have likely started dialysis (had they chosen it).

### Predictors of survival in older patients

Based on presumed clinical significance, univariate Cox regression identified age, CCI score and treatment modality (RRT vs MCM) as significant predictors of survival at 3 years from the time of choice in patients. Poverty score and sex were not associated with survival in this cohort.

A multivariable Cox regression model of age, sex, comorbidity and poverty was used to estimate the independent association of each variable on hazards. Higher comorbidity as well as being older were associated with lower survival despite modality choice not being in the model but poverty was not significant and was removed from the model (Table 4). On adding choice to the model, higher comorbidity and older age remained associated with higher mortality but choosing RRT over MCM also predicted survival. Survival remained significantly better in patients choosing RRT over MCM even in a subset of patients over 80 years of age with comorbidity scores greater than 4 (p = 0.007, HR 1.555 (95%CI: 1.129–2.142)).

**Table 4:** Stepwise Multivariate Cox proportional hazards model for survival in patients aged ≥70 years old using the time of modality choice as the starting point in survival calculation. Poverty was not significant and was removed. Choice and then Frailty (at the expense of CCI) added to the model. The last model was on a subset of the frailest patients (CFS≥6).

All ≥ 70; n = 1 216	β	P-value	Hazard Ratio	95% CI for Hazard Ratio
Sex (Female = 1)	-0.09	0.31	0.91	0.77–1.09
CCI (per unit increase)	0.08	0.002	1.08	1.03–1.14
Poverty (per unit increase)	0.00	0.85	1.00	1.00–1.00
Age (per year increase)	0.07	<0.001	1.07	1.06–1.09
All ≥ 70; n = 1 216	*Choice in model*			
Sex (Female = 1)	-0.15	0.10	0.86	0.73–1.03
CCI (per unit increase)	0.07	0.007	1.07	1.02–1.12
Age (per year increase)	0.05	<0.001	1.05	1.03–1.07
Choice (MCM = 1)	0.60	<0.001	1.83	1.51–2.22
≥ 70 with CFS; n = 486	*Frailty in model*			
Sex (Female = 1)	-0.42	0.003	0.66	0.50–0.87
CCI (per unit increase)	0.02	0.63	1.02	0.94–1.10
Age (per year increase)	0.04	0.001	1.05	1.02–1.07
Choice (MCM = 1)	0.55	0.001	1.73	1.27–2.36
CFS (per unit increase)	0.25	<0.001	1.29	1.15–1.45
≥ 70 years, CFS ≥ 6; n = 140	*Frailest patients only*			
Sex (Female = 1)	-0.09	0.70	0.91	0.58–1.44
CCI (per unit increase)	0.01	0.84	1.01	0.88–1.17
Age (per year increase)	0.08	0.001	1.08	1.03–1.14
Choice (MCM = 1)	0.18	0.52	1.20	0.69–2.06

On adding CFS to the model (n = 486)–sex, age, choice and CFS all predicted 3-year survival while comorbidity had no significant effect. However, when only patients with CFS ≥ 6 (at least moderate frailty, needing assistance with all outside activities or unable to undertake outside activities without support) were included in the analysis (n = 140, MCM 83 and HD 57), only age rather than the choice of RRT vs MCM significantly affected survival.

## Discussion

In this analysis we have described the demographics, choices and outcomes of a large population of older pre-dialysis patients who are a heterogeneous mix of patients with varying degrees of comorbidity, frailty and poverty. To our knowledge this is the largest study of this type that currently exists [5, 11, 16, 25, 26]. Our analysis has identified a number of novel findings that may help inform the pre-dialysis shared decision-making process in this population.

Age is a strong predictor of choice with the likelihood of choosing RRT falling with increasing age. The majority of patients over the age of 80 opt for MCM. Unlike other studies, comorbidity does not appear to predict patient choices [26–28]. This may be explained by our observation that both comorbidity scores and levels of poverty fall with increasing age, hinting at a survivor bias whereby younger patients with higher levels of poverty and higher levels of comorbidity are more likely to die than to survive to an age where they develop advanced kidney disease. This is a novel finding which has not been reported in other studies [26, 29]. The generalisability of results from our overwhelmingly white population with its local socioeconomic and geographical characteristics is uncertain. Timing of presentation to renal services, nature of renal disease and its progression and cultural attitudes to treatment may differ in other populations and impact on who is seen for pre-dialysis education.

In contrast to comorbidity, frailty is a strong predictor of whether patients opt for MCM rather than RRT. Having multiple diagnosed health problems does not necessarily equate to the complex syndrome of frailty [30]. Given that increasing frailty impacts on the ability to perform daily activities and to leave the home independently [21], it is likely that frailty limits a patients’ perceived ability to cope with the burdensome nature of RRT.

The novel finding that level of poverty influence the likelihood of choosing MCM (independent of age and comorbidity) may reflect economically driven differences in preferences/values and expectations so that more affluent patients may chose a more active form of therapy [31]. It may also reflect underlying physician bias (either conscious or unconscious). This will require further analysis in future qualitative studies [32].

Our study demonstrates that once patients have made an initial choice, they rarely change their minds about therapy with hardly any patients switching their choice from HD to PD or from MCM to HD. This reflects the previously documented patient preference for maintaining the status quo, in spite of patients often regretting their choices [13, 33]. Our model of shared decision making is a dynamic patient centred dialogue and the aim is to prioritise the goals and aims of the patient rather than to focus on the disease itself. It is a continuous process and it may be an oversimplification to define a time of choice particularly in the context of caring for frail older patients in whom more complex shared decision making models are being developed [34].

In terms of survival, our study is the largest to date that has analysed survival from the time of patient choice [11]. In a centre with a standardised pre-dialysis pathway this is an important time-point that should support the decision-making process for the patient. Over half of all patients aged over 70 counselled about RRT die before ever starting RRT. As with other published cohorts, those choosing RRT live longer than those choosing MCM from the time of choice [27, 28]. The two groups are, however, significantly different in terms of age and frailty, which is an inherent problem with retrospective analyses of patient outcomes. In spite of this limitation we believe that our data provides important information that will support the shared decision-making process by enabling more bespoke patient centred discussions about realistic outcomes.

Since our data contains a large number of patients who have chosen MCM (the largest studied cohort to date) we have been able to better characterise the outcome of these patients. It is notable that over 70% of patients who opt for MCM die with an eGFR > 9ml/min (median dialysis initial eGFR in our unit) suggesting that most patients who chose MCM never reach a point where they would have likely started dialysis (had they chosen it). Choosing MCM seems to adversely affect survival independent of age, comorbidity and poverty—so that even in patients over the age of 80 with a comorbidity score > 4, choosing MCM rather than dialysis is associated with poorer survival. Whilst, as in other studies of patients making pre-dialysis choices, comorbidity predicts outcomes of patients with advanced kidney disease [27, 29], our study suggests frailty appears to be a better predictor. Frailty has been shown to independently predict outcomes in patients already on dialysis [35]. We have shown that across all age ranges once patients have a frailty score of at least 6 (which describes a dependant cohort) their survival seems unaffected by whether they chose dialysis or not. The impact of frailty on patient outcomes in advanced kidney disease is increasingly recognised however an ongoing debate persists on how frailty is most effectively assessed [36–40].

We believe our findings should further support the routine prospective assessment and recording of frailty in all pre-dialysis patients. Such data may help predict patients who can avoid burdensome pre-dialysis planning.

Survival analysis in patients with ESRD whether from the time of choice or RRT start is exposed to bias around clinician practice [28, 41, 42]. Retrospective analyses around RRT decisions and outcomes are inherently flawed due to selection bias and residual confounding due to unmeasured variables [43]. We cannot exclude the possibility that some older comorbid patients are never referred to nephrology services and managed conservatively by other health care providers. However, the lack of local anecdotal encounters suggests that this is not the case.

## Conclusions

Our study has demonstrated that many factors including frailty and socio-economic status influence patient choices and outcomes. Many older patients who receive pre-dialysis education die before ever having RRT and most who choose MCM die before developing end stage kidney failure. Furthermore, although high comorbidity is associated with lower survival, age and frailty appear to be better predictors of patient choices and outcomes. We believe that routine collection of frailty data will eventually lead to more robust predictive tools that will better inform the pre-dialysis shared decision-making process.

## Supporting information

S1 Fig Pre-dialysis pathway. (TIF)

S2 Fig Domains of the pre-dialysis discussion. (TIF)

S1 Data (XLSX)
