# Supplementary figures and images for: Exploring the choices and outcomes of older patients with advanced kidney disease
Source: PLoS One. 2020 Jun 10;15(6):e0234309. doi: 10.1371/journal.pone.0234309 (PMC7286495; doi:10.1371/journal.pone.0234309)

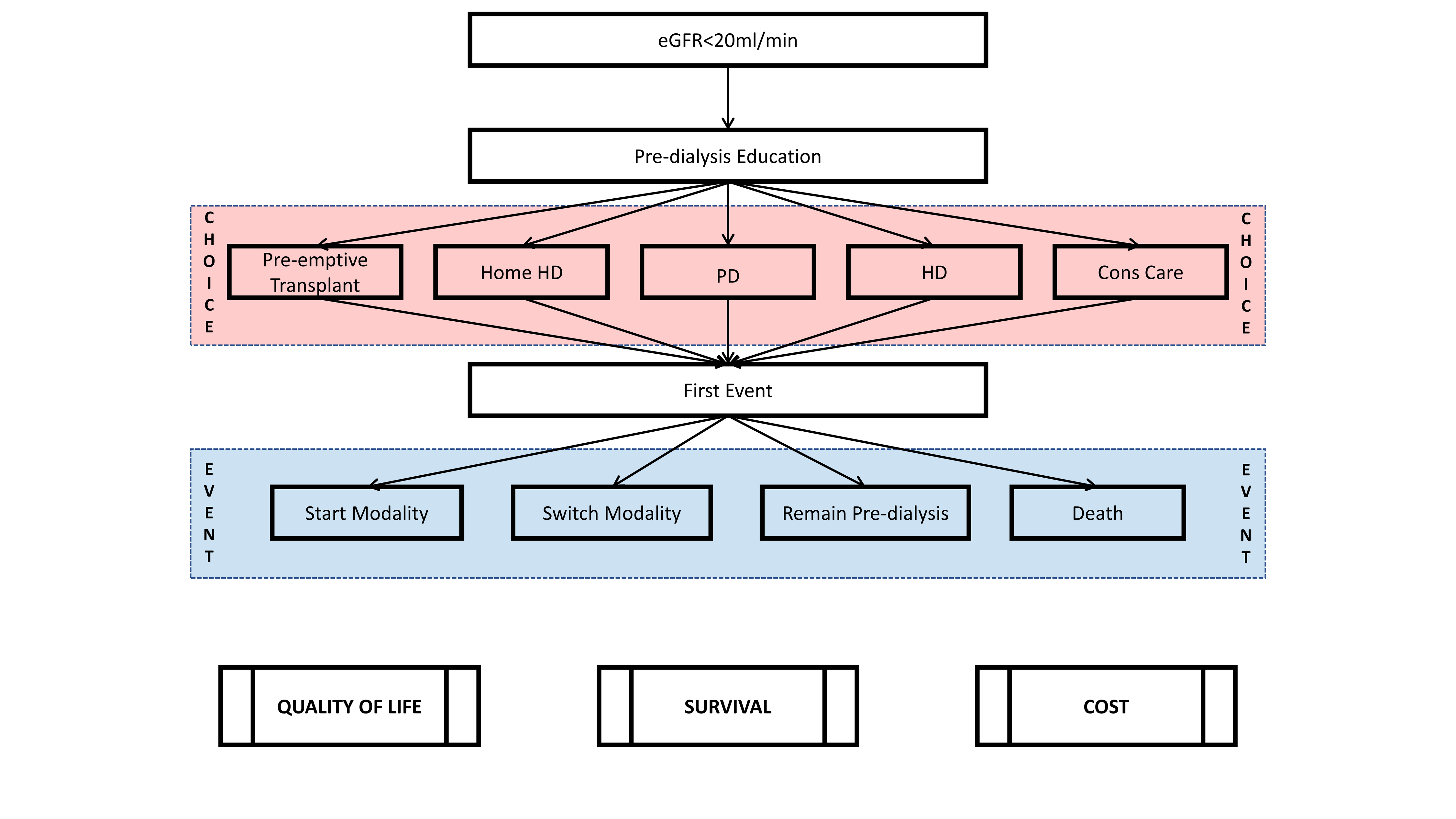

Supplement: S1 Fig — (TIF) [file pone.0234309.s001.tif]

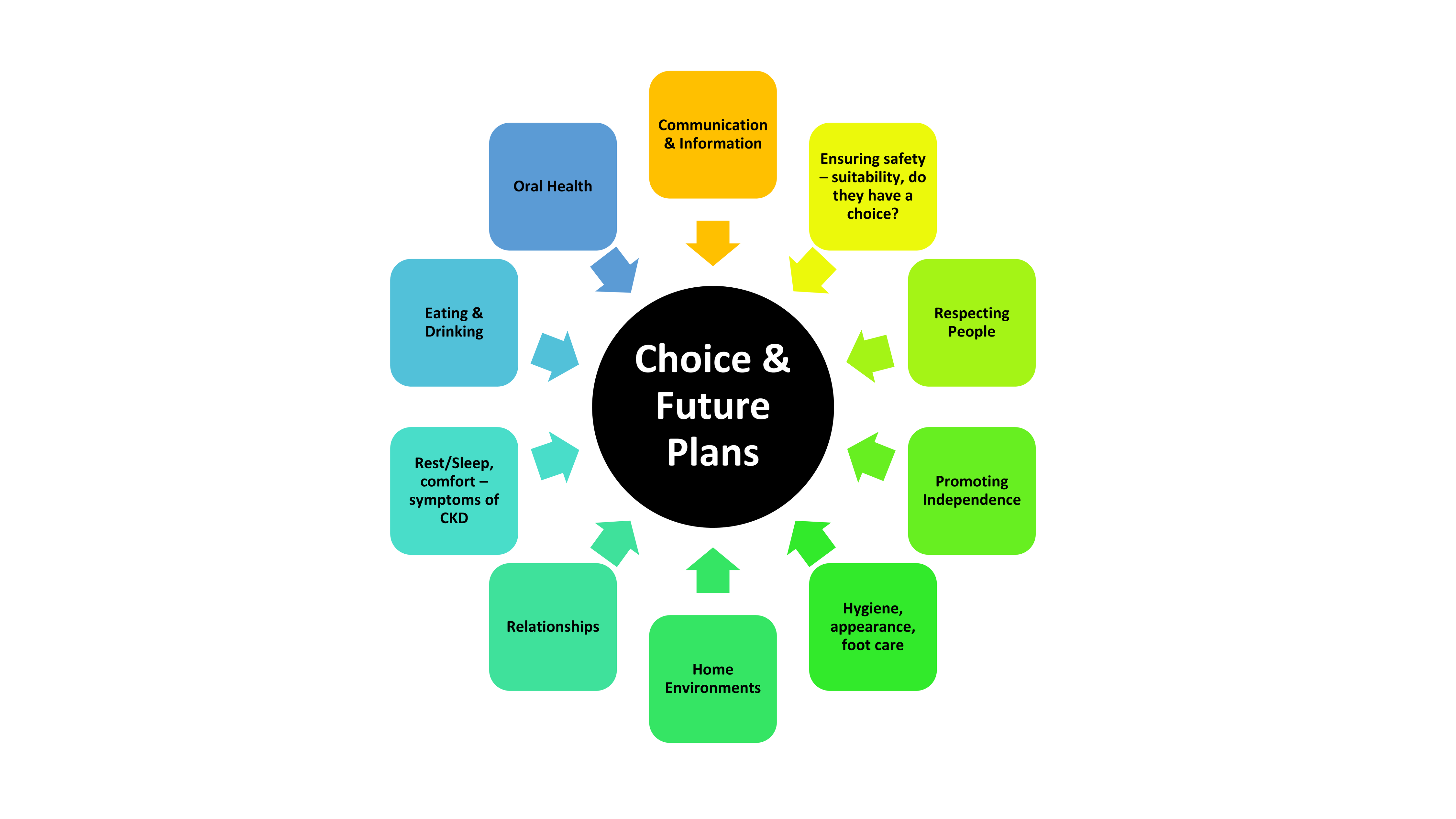

Supplement: S2 Fig — (TIF) [file pone.0234309.s002.tif]
